# Determinants of micronutrient supplementation during pregnancy among women in three sub-Saharan African countries: a multilevel logistic regression model

**DOI:** 10.3389/fgwh.2024.1449259

**Published:** 2024-10-25

**Authors:** Enyew Getaneh Mekonen, Alebachew Ferede Zegeye, Belayneh Shetie Workneh, Mohammed Seid Ali, Almaz Tefera Gonete, Tewodros Getaneh Alemu, Tadesse Tarik Tamir, Berhan Tekeba, Mulugeta Wassie, Alemneh Tadesse Kassie

**Affiliations:** ^1^ Department of Surgical Nursing, School of Nursing, College of Medicine and Health Sciences, University of Gondar, Gondar, Ethiopia; ^2^ Department of Medical Nursing, School of Nursing, College of Medicine and Health Sciences, University of Gondar, Gondar, Ethiopia; ^3^ Department of Emergency and Critical Care Nursing, School of Nursing, College of Medicine and Health Sciences, University of Gondar, Gondar, Ethiopia; ^4^ Department of Pediatrics and Child Health Nursing, School of Nursing, College of Medicine and Health Sciences, University of Gondar, Gondar, Ethiopia; ^5^ School of Nursing, College of Medicine and Health Sciences, University of Gondar, Gondar, Ethiopia; ^6^ Department of Clinical Midwifery, School of Midwifery, College of Medicine and Health Sciences, University of Gondar, Gondar, Ethiopia

**Keywords:** micronutrient intake, pregnant women, DHS, sub-Saharan Africa, multilevel analysis

## Abstract

**Background:**

Poor maternal nutrition during pregnancy is a common cause of poor maternal and infant outcomes. Micronutrient deficiencies are common among pregnant women in low- and middle-income countries, including sub-Saharan Africa. Pregnant women are recommended to take micronutrients like iron or folic acid and deworming medication during pregnancy. Therefore, this study was conducted to assess micronutrient intake and its associated factors among pregnant women in three countries using the most recent demographic and health survey.

**Methods:**

We used data from the most recent demographic and health surveys, which were carried out between 2019 and 2022 in three sub-Saharan African countries. The study included a weighted sample of 13,568 reproductive-age women who had given birth within the five years prior to the survey. Utilizing multilevel logistic regression, the factors associated with the dependent variable were identified. Model comparison and fitness were assessed using the deviance (-2LLR), likelihood ratio test, median odds ratio, and intra-class correlation coefficient. Ultimately, factors were deemed statistically significant if they had a *p*-value less than 0.05.

**Results:**

The pooled prevalence of micronutrient intake among pregnant women during pregnancy of last birth was 77.56% (95% CI: 76.85%–78.25%). Factors like age [AOR = 1.78; 95% CI (1.14, 2.77)], educational status [AOR = 1.49; 95% CI (1.23, 1.79)], marital status [AOR = 0.66; 95% CI (0.58, 0.75)], working status [AOR = 1.17; 95% CI (1.01, 1.34)], media exposure [AOR = 1.20; 95% CI (1.05, 1.38)], preceding birth interval [AOR = 1.17; 95% CI (1.01, 1.34)], number of ANC visits [AOR = 1.65; 95% CI (1.29, 2.10)], and residence [AOR = 1.19; 95% CI (1.03, 1.37)] were significantly associated with micronutrient intake among pregnant women.

**Conclusions:**

More than three-fourths of the study subjects were micronutrient supplemented during their pregnancy. Improving women's education, disseminating nutrition information through media, providing more attention to young pregnant women who live in rural areas, increasing the number of ANC visits, and women's empowerment are strongly recommended.

## Background

Maternal undernutrition is a common public health problem and a crucial determinant of poor perinatal outcomes in sub-Saharan Africa (SSA) (1). Poor maternal nutrition during pregnancy is a common cause of poor maternal and infant outcomes, including low birth weight (2), maternal anemia (3), maternal mortality (4), preterm delivery (3), and neural tube defects (NTD) (5) in developing countries. Micronutrient deficiency during pregnancy also impacts the mother's well-being and the growth of fetal organs (6). Pregnant women are principally vulnerable to the effects of micronutrient malnutrition as a result of the high requirements of the growing fetus, placenta, and maternal tissues (7). An inability to fulfill the increased requirements results in potentially adverse outcomes for the mother and the fetus (8).

The physiological changes during pregnancy are usual adaptations to cultivate the developing fetus and prepare the mother for normal labor and delivery, which result in changing nutritional needs (9). Immediately after conception, these physiological changes begin and affect body systems, including the endocrine, cardiovascular, hematological, gastrointestinal, skeletal, and respiratory systems (10). Pregnancy also places irreplaceable demands on a woman's body, with extra energy and an improved intake of nutrients needed to help support ideal fetal growth (11). Appropriate nutrition is essential for normal neurological development, which is rapid during the first thousand days of life, with changes occurring from post-conception until 24 months of age (12). Pregnant women are advised to consume vegetable, fruit, and whole grain-rich foods and to take a regular vitamin and mineral supplement to assure adequate intake of iron and folic acid to meet nutritional needs (13).

Micronutrients, including essential vitamins and minerals, have important roles in the health of pregnant women and the growing fetus (14). Micronutrients can be used as co-factors and coenzymes for the metabolism of nutrients and are useful in preventing different diseases in pregnant women (15). Micronutrient demands increase more than dietary energy demands during pregnancy (16). During pregnancy, iron requirements exceed more than two times the pre-gestational period as a result of feto-placental requirements and the expansion of maternal red blood cells. This results in an increase in iron demand of 30 mg/day during pregnancy, which increases from around 0.8 mg/day in the 1st trimester to 4–5 mg/day in the 2nd trimester and more than 6 mg/day in the 3rd trimester (17). The daily recommended allowance of oral ferrous iron for pregnant women is 27 mg (15). For all women of childbearing age, a daily supplementation of folic acid 400–800 μg is suggested in order to decrease the risk of neural tube defects in the fetus from two to three months before and after conception, respectively (18). An increased dosage of 4–5 mg/d is required in the case of mothers with previous children with NTD, use of anti-seizure medications, and pre-existing chronic conditions like diabetes mellitus, which increase the risk of NTD or folate deficiency (19).

Micronutrient deficiencies are common among pregnant women in low- and middle-income countries, including SSA, due to a lack of diet diversity and food fortification (20). The most common known micronutrient deficiency that causes anemia is maternal iron deficiency, which is estimated to affect 40% of pregnant women globally, with the highest prevalence in Africa (46%) (21). During pregnancy, insufficient stores or intake of micronutrients could cause anemia, hypertension, different labor complications, death to the mother and stillbirth, pre-term delivery, intrauterine growth retardation, congenital defects, reduced immune competence, and irregular organ development in the fetus (22). Micronutrient deficiencies can also lead to maternal morbidity and mortality since they are vital for fetal development (15). According to the World Health Organization (WHO), pregnant women are recommended to take iron or folic acid at least for 90 days and to take deworming medication (drugs for intestinal parasites) during pregnancy (23). However, pregnant women in SSA countries universally lack nutrient-rich diets that result in deficiencies of micronutrients (24). A previous study conducted in East African countries showed that the pooled prevalence of micronutrient intake was 36.07% (25). However, there has been no study conducted regarding micronutrient intake status and associated factors among pregnant women in SSA using the most recent (2019–2022) demographic and health survey (DHS) data. Therefore, the findings of this study will inform policymakers and program managers that work on maternal child health to design appropriate interventions to improve the intake of micronutrients among pregnant women.

## Methods and materials

### Data sources, sampling, and study populations

The most recent DHS data from the three SSA nations of Tanzania (2022), Kenya (2022), and Gabon (2019–21) is used in this study. A multilevel mixed effects analysis was done. Every five years, the community-based survey (DHS) is carried out to generate updated demographic and health-related data. The source population for the study consisted of all pregnant women in the three SSA nations. The study population for this research consisted of pregnant women who were residing in the randomly chosen enumeration areas of each country throughout the survey year. In order to determine the micronutrient consumption status and related determinants among pregnant women in three SSA countries, the data were appended. Different datasets, such as those for children, men, women, births, and households, are included in the survey for each nation. In this investigation, the individual record (IR) file was utilized. The DHS is a national survey that is mostly conducted in low- and middle-income nations every five years. In order to enable cross-country comparison, consistent techniques are used for sampling, questionnaires, data collection, cleaning, coding, and analysis (26). The study included a weighted sample of 13,568 women in the reproductive age range who fully responded to all factors of interest (Table 1). The DHS uses a two-stage, stratified sampling method (27). The first step is creating a sample frame, which is a list of enumeration areas (EAs) or primary sampling units (PSUs) that encompass the entire nation. This list is typically created using the most recent national census that is available. The systematic sampling of the households included in each cluster, or EA, is the second step. The DHS guideline provides more details on survey sampling techniques (28).

**Table 1:** Sample size for micronutrient intake status and associated factors among pregnant women in three sub-Saharan African countries.

Country	Year of survey	Weighted sample (*n*)	Weighted sample (%)
Gabon	2019–21	3,982	29.35
Kenya	2022	4,838	35.66
Tanzania	2022	4,748	34.99
Total sample size		13,568	100

### Variables of the study

#### Dependent variable

Micronutrient intake status.

For this study, women who took tablets or syrups of iron or folic acid at least for 90 days or took deworming medication during pregnancy of last birth were considered micronutrient supplemented (1), and those who didn't were micronutrient not supplemented (0). This information was self-reported by the woman herself.

#### Independent variables

Independent variables from two sources (variables at the individual and community levels) were taken into account for this analysis because DHS data are hierarchical. Individual-level variables: respondents’ age (15–19, 20–25, 26–34, and 35–49 years), educational status (no education, primary, secondary & higher), household wealth index (poor, middle, rich), current marital status (married, unmarried), birth order (1–4, 5–9, 10+), birth interval in months (short interval; <24 months, long interval; ≥24 months), distance to health facility (big problem, not a big problem), working status (working, not working), media exposure (yes, no), number of ANC visits (no visit, <4 visits, ≥4 visits), household size (≤5, >5), and sex of household head (male, female). Community-level variables**:** place of residency (rural or urban), community-level media exposure (low or high), community-level maternal literacy (low or high), and community-level maternal poverty (low or high).

### Data management and analysis

STATA/SE version 14.0 statistical software was used to clean, recode, and analyze data that was taken from the most recent DHS data sets. A sample weight was used to control non-responses and sampling mistakes. The presentation of the variables at the individual and community levels was done using descriptive statistics. Women are nested within households, and households are nested within clusters, according to the cluster-based organization of the DHS data's variables. In order to use the conventional logistic regression model, the presumptions of independent observations and equal variance across clusters were broken. This suggests that accounting for between-cluster effects requires the use of a complex model. To ascertain the variables associated with micronutrient consumption, multilevel logistic regression was applied. The null model (outcome variable only), model I (only individual-level variables), model II (only community-level variables), and model III (both individual and community-level variables) are the four models that multilevel mixed effect logistic regression uses. The null model, which is devoid of independent variables, was employed to examine the variation in micronutrient intake throughout the cluster. Evaluations were conducted on the relationships between the outcome variable (Model I) and the factors at the individual and community levels (Model II). The connection between the community- and individual-level variables and the outcome variable (micronutrient intake) was fitted simultaneously in the final model, or Model III. Through the use of the intra-class correlation coefficient (ICC) and proportional change in deviance (PCV), the magnitude of the clustering effect and the extent to which community-level factors explain the unexplained variance of the null model were assessed. The best-fitting model was determined to be the one with the lowest deviance. Lastly, variables were reported as statistically significant variables linked with micronutrient consumption if they had a *p*-value of less than 0.05 and an adjusted odds ratio (AOR) with a 95% confidence interval (CI). A variance inflation factor (VIF) lying within acceptable bounds of 1–10 was used to test for multi-collinearity amongst covariates, showing the lack of significant collinearity among explanatory variables.

### Random effects

The measures of variation of the outcome variable, or random effects, were estimated using the PCV, ICC, and median odds ratio (MOR). The difference between clusters was measured using the ICC and PCV. Taking clusters as a random variable, the ICC reveals that the variation of micronutrient intake between clusters is computed as follows: $\;\;\mathrm{ICC}=\frac{\mathrm{VC}}{\mathrm{VC}+3.29}\times 100\text{\%}$. The MOR is the median value of the odds ratio between the area of the highest risk and the area of the lowest risk for micronutrient intake when two clusters are randomly selected, using clusters as a random variable; MOR = *e*^0.95√VC^. In addition, the PCV demonstrates the variation in micronutrient intake explained by factors and is computed as: $\mathrm{PCV}=\frac{\mathrm{Vnull}-\mathrm{VC}}{\mathrm{Vnull}}\times 100\text{\%}$; where Vnull = variance of the null model and VC = cluster level variance (29–31). The association between the likelihood of micronutrient intake and individual and community-level independent variables was estimated by the fixed effects (32–34).

### Ethical approval and consent to participate

Before starting the study, permission was given to download and use the data from http://www.dhs.program.com. The Institution Review Board of the DHS Program, ICF International, granted ethical approval. The Institution Review Board gave its approval to the DHS public-use data set procedures. Names of persons or household addresses were not included in the data files, and identifiers for respondents, households, or sample communities were prohibited in any manner. There are no labels on the data file that list the names or locations of any of the EAs's numbers. Since the study's data set was made available to the public, neither patients nor members of the public were participating.

## Results

### Individual and community-level characteristics of pregnant women

A total of 13,568 pregnant women were included in this study. The mean age of pregnant women was 29.30 ± 0.04 years, and 40% of them fall in the age range of 26–34 years. Nearly half (49.88%) of the pregnant women had completed secondary and higher education, and 51.44% of them were currently married. More than half (53.42%) of pregnant women had work, and 76.50% of them had media exposure. Nearly 48% of pregnant women had poor socioeconomic status. About 75.97% of pregnant women reported a birth order of 1–4, and 83.04% of them had a long birth interval. More than two-thirds (69.70%) of pregnant women attended 4+ ANC visits during pregnancy. Distance to a health facility was not a big problem for 63.94% of pregnant women. More than half (51.21%) of pregnant women had a household size greater than five, and the sex of the household head was male for 71% of them. More than half (56.01%) of the study subjects were from rural areas, and 50.74% of the community had low media exposure. More than half (52.44%) and (57.95%) of pregnant women had high community poverty and community literacy, respectively (Table 2).

**Table 2:** Individual and community-level characteristics of pregnant women, pooled data from three sub-Saharan African countries.

Variables	Category	Frequency (*n*)	Percentage (%)
Age	15–19 years	987	7.27
	20–25 years	4,138	30.50
	26–34 years	5,430	40.02
	35–49 years	3,013	22.21
Educational status	No formal education	1,922	14.17
	Primary	4,877	35.95
	Secondary and higher	6,769	49.88
Marital status	Married	6,979	51.44
	Unmarried	6,589	48.56
Working status	Not working	6,318	46.58
	Working	7,246	53.42
Media exposure	Yes	10,380	76.50
	No	3,188	23.50
Wealth index	Poor	6,471	47.70
	Middle	2,469	18.20
	Rich	4,628	34.10
Birth order	1–4	10,307	75.97
	5–9	3,047	22.46
	10+	214	1.57
Distance to health facility	Big problem	4,413	36.06
	Not a big problem	7,825	63.94
Birth interval	Short	1,736	16.96
	Long	8,501	83.04
Number of ANC visits	No visit	646	4.76
	<4 visits	3,465	25.54
	≥4 visits	9,457	69.70
Household size	≤5	6,620	48.79
	>5	6,948	51.21
Sex of the household head	Male	9,577	70.59
	Female	3,991	29.41
Type of place of residence	Urban	5,969	43.99
	Rural	7,599	56.01
Community media exposure	Low	6,684	49.26
	High	6,884	50.74
Community poverty	Low	6,453	47.56
	High	7,115	52.44
Community literacy	Low	5,705	42.05
	High	7,863	57.95

### Micronutrient intake status among pregnant women

**Figure 1:**
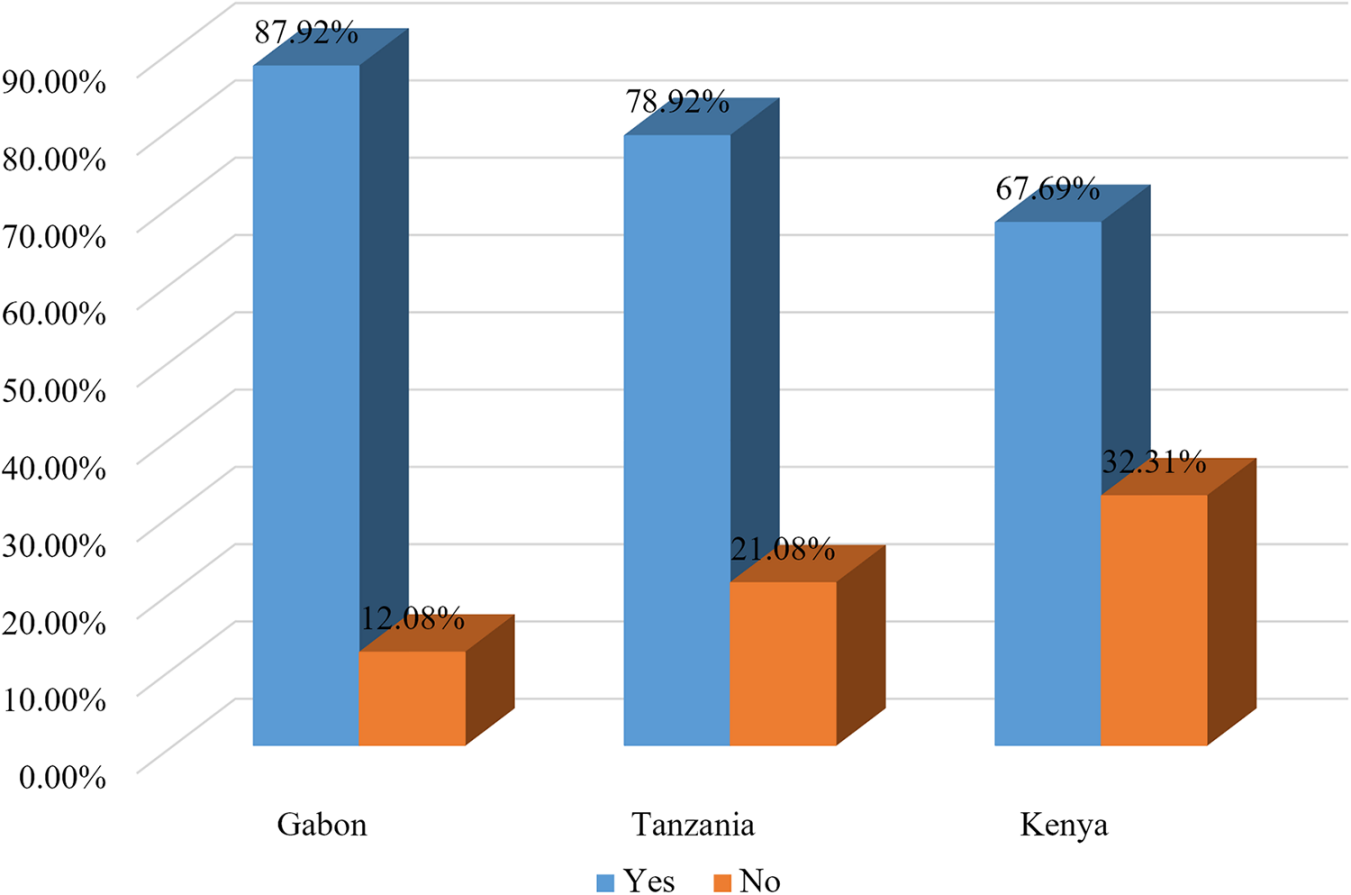
Variation of micronutrient intake among pregnant women by countries.

In the current study, 77.56% (95% CI: 76.85%–78.25%) of pregnant women took micronutrients during the pregnancy of their last birth (took tablets or syrup of iron or folic acid at least for 90 days or deworming medication during the pregnancy of their last birth). Women from Kenya had the lowest prevalence of micronutrient intake (67.69%). On the other hand, women from Gabon had the highest prevalence of micronutrient intake (87.92%) (Figure 1).

### Random effects (measures of variation) and model fitness

To determine whether the data supported the decision to assess randomness at the community level, a null model was used. According to findings from the null model, there were significant differences in micronutrient intake status between communities, with a variance of 0.614 and a *P* value of <0.001. The variance within clusters contributed 84.28% of the variation in micronutrient intake status, while the variance across clusters was responsible for 15.72% of the variation. In the null model, the odds of micronutrient intake status differed between higher and lower-risk clusters by a factor of 2.11 times. The intra-class correlation value for Model I indicated that 13.60% of the variation in micronutrient intake status accounts for the disparities between communities. Then, with the null model, we used community-level variables to generate Model II. According to the ICC value from Model II, cluster variations were the basis for 9.87% of the differences in micronutrient intake status. In the final model (model III), which attributed approximately 21% of the variation in the likelihood of micronutrient intake status to both individual and community-level variables, the likelihood of micronutrient intake status varied by 1.93 times across low and high micronutrient intake (Table 3).

**Table 3:** Model comparison and random effect analysis for micronutrient intake status among pregnant women in three sub-Saharan African countries.

Parameter	Null model	Model I	Model II	Model III
Variance	0.6137628	0.5179457	0.5531778	0.4850132
ICC	15.72%	13.60%	14.39%	12.85%
MOR	2.11	1.89	2.03	1.93
PCV	Reference	15.61%	9.87%	20.98%
Model fitness				
LLR	−7,070.2209	−4,746.152	−6,982.9281	−4,722.2513
Deviance	14,140.4418	9,492.304	13,965.8562	9,444.5026

ICC, intra cluster correlation; LLR, log-likelihood ratio; MOR, median odds ratio; PCV, proportional change in variance.

### Individual and community-level factors associated with micronutrient status

In the final fitted model of multivariable multilevel logistic regression, women's age, educational status, marital status, working status, media exposure, birth interval, numbers of ANC visits attended during pregnancy, type of place of residence, and community media exposure were factors significantly associated with micronutrient intake among pregnant women.

The odds of micronutrient intake were 1.56 and 1.78 times higher among pregnant women aged 26–34 years and 35–49 years compared with women aged 15–19 years, respectively [AOR = 1.56; 95% CI (1.01, 2.39)] and [AOR = 1.78; 95% CI (1.14, 2.77)]. The odds of micronutrient intake were 1.36 and 1.49 times higher among pregnant women who completed primary and secondary education compared with those who had no formal education [AOR = 1.36; 95% CI (1.15, 1.59)] and [AOR = 1.49; 95% CI (1.23, 1.79)]. Unmarried women are 34% less likely to have micronutrient intake compared with married women [AOR = 0.66; 95% CI (0.58, 0.75)]. Pregnant women with any work were 1.17 times more likely to have micronutrient intake than those who were not working [AOR = 1.17; 95% CI (1.01, 1.34)]. Similarly, pregnant women who had media exposure were 1.20 times more likely to have micronutrient intake compared with their counterparts [AOR = 1.20; 95% CI (1.05, 1.38)].

Pregnant women with a long birth interval were 1.17 times more likely to have micronutrient intake compared with their counterparts [AOR = 1.17; 95% CI (1.01, 1.34)]. Pregnant women who attended ≥4 ANC visits during pregnancy were 1.65 times more likely to have micronutrient intake compared with those who didn't attend ANC visits [AOR = 1.65; 95% CI (1.29, 2.10)]. The odds of micronutrient intake were 1.19 times higher among pregnant women from urban areas than those from rural areas [AOR = 1.19; 95% CI (1.03, 1.37)]. Pregnant women from a community with low media exposure were 32% less likely to have micronutrient intake compared with pregnant women from a community with high media exposure [AOR = 0.68; 95% CI (0.57, 0.82)] (Table 4).

**Table 4:** Multivariable multilevel logistic regression analysis of individual and community-level factors associated with micronutrient intake among pregnant women in three SSA countries.

Variables	Category	Model I AOR (95% CI)	Model II AOR (95% CI)	Model III AOR (95% CI)
Age	15–19 years	1		1
	20–25 years	1.31 (0.85, 2.01)		1.27 (0.82, 1.94)
	26–34 years	1.59 (1.04,2.44)*		1.56 (1.01,2.39)*
	35–49 years	1.81 (1.16,2.83)*		1.78 (1.14,2.77)*
Educational status	No education	1		1
	Primary	1.26 (1.07,1.47)*		1.36 (1.15,1.59)*
	Secondary/higher	1.37 (1.15,1.64)*		1.49 (1.23,1.79)*
Marital status	Married	0.62 (0.55,0.70)*		0.66 (0.58,0.75)*
	Unmarried	1		1
Working status	Not working	1		1
	Working	1.15 (1.03,1.29)*		1.17 (1.01,1.34)*
Media exposure	Yes	1.12 (0.98, 1.28)		1.20 (1.05,1.38)*
	No	1		1
Wealth index	Poor	1		1
	Middle	1.06 (0.91, 1.23)		1.06 (0.91, 1.24)
	Rich	1.14 (0.98, 1.33)		1.12 (0.94, 1.32)
Birth order	1–4	1		1
	5–9	0.93 (0.80, 1.08)		0.91 (0.78, 1.06)
	10+	0.80 (0.55, 1.17)		0.79 (0.54, 1.15)
Distance to health facility	Big problem	1		1
	Not a big problem	0.88 (0.78,0.99)*		0.90 (0.80, 1.01)
Birth interval	Short	1		1
	Long	1.15 (1.00,1.32)*		1.17 (1.01,1.34)*
Number of ANC visits	No visit	1		1
	<4 visits	0.58 (0.46,0.75)*		0.61 (0.47,0.78)*
	≥4 visits	1.60 (1.25,2.04)*		1.65 (1.29,2.10)*
Household size	≤5	0.90 (0.80, 1.02)		0.89 (0.79, 1.01)
	>5	1		1
Sex of the household head	Male	1		1
	Female	0.92 (0.81, 1.05)		0.92 (0.81, 1.05)
Place of residence	Rural		1	1
	Urban		1.57 (1.41,1.75)*	1.19 (1.03,1.37)*
Community media exposure	Low		1	1
	High		0.72 (0.61,0.85)*	0.68 (0.57,0.82)*
Community poverty	High		1	1
	Low		1.09 (0.94, 1.25)	0.97 (0.82, 1.15)
Community literacy	Low		1	1
	High		1.04 (0.88, 1.23)	0.94 (0.78, 1.13)

Model I: only individual-level variables; Model II: only community-level variables; Model III: both individual and community-level variables.

* Statistically significant at *p*-value <0.05.

## Discussion

This study was conducted to investigate the pooled prevalence and its determinants of micronutrient intake among pregnant women in three SSA countries, which is essential to lessening the problem of micronutrient deficiency among women of reproductive age. In the current study, the pooled prevalence of micronutrient intake in three SSA countries was 77.56% (95% CI: 76.85%–78.25%). This finding was higher than studies conducted in Ethiopia (44.3%) (35), Morocco (33.3%) (36), Ghana (46.1%) (37), and East African countries (36%) (25). The possible justification for this difference might be due to socio-cultural and time differences. The current study uses the most recent DHS data from three SSA countries that might contribute to the high intake of micronutrients among pregnant women. Some previous studies were also conducted among women of reproductive age groups in a single country and zone that might not be representative at the national and continent levels. In this study, about 54.22% (95% CI: 53.44%, 55.00%) of pregnant women in the three SSA countries took deworming medication during the pregnancy of their last birth. This finding was lower than a study conducted in Benin (65%) (38). This discrepancy might be due to differences in the socio-demographic characteristics of the study subjects. On the other hand, the current finding was higher than studies conducted in 26 sub-Saharan African countries (50.7%) (39) and Cameroon (29.8%) (40). This could be attributed to differences in the methodology of the studies (all reproductive-age women vs. married women, as well as included countries and study areas).

Young pregnant women are at risk of low micronutrient intake, in which pregnant women aged 26–34 years and 35–49 years were more likely to take micronutrients. This finding was consistent with studies conducted in Ethiopia (35), east African countries (25), and South Africa (41). This might be due to the difference in diets between young adults and older people. In addition, women with delayed first pregnancies may differ from younger women of comparable education and social class in a range of health-related activities. Older women might have better diets than younger women and are more likely to have planned pregnancies that motivate them to change to a healthier diet (42). Similarly, pregnant women who completed primary and secondary education were more likely to take micronutrients compared with those who had no formal education. This finding was consistent with studies conducted in Ethiopia (43), Bangladesh (44), east African countries (25), and Kenya (45). This might be due to the fact that women with a higher educational status have better food habits than those with a lower education. A study in Japan also supports this finding, in which higher educational status is associated with higher intakes of fatty acids, dietary fiber, protein, cholesterol, calcium, potassium, magnesium, iron, vitamin A, D, E, and C, and folic acid (46). Unmarried women were less likely to take micronutrients compared with married women. This finding was supported by studies conducted in Ethiopia (47) and East African countries (25). This could be due to the enhancement of family income and wealth associated with marriage. Married women are also more food secure as they get social support and other noneconomic resources from their partner. A study conducted in Ghana indicated a higher prevalence of food insecurity among unmarried women than among those who are married (48). In addition, pregnant women with any work were more likely to take micronutrients than those who were not working. A study conducted in east African countries (25) reported a similar finding. This might be due to the fact that those who have jobs have a consistent income, which increases their chances of accessing nutrition. Media exposure was another determinant of micronutrient intake. Pregnant women who had media exposure were more likely to take micronutrients compared with their counterparts. This finding was in agreement with studies conducted in Ethiopia (47) and east African countries (25). The possible justification might be that exposure to media increases access to information about adequate intake of micronutrients by pregnant women. Media exposure plays a substantial role in raising awareness about critical maternal health issues, including micronutrient supplementation during pregnancy (49). Media exposure is also correlated with the use of maternal healthcare services including prenatal care services (50).

Likewise, pregnant women with a long birth interval (≥24 months) were more likely to take micronutrients. This finding was in agreement with studies conducted in Ethiopia (51) and Nigeria (52). This might be due to the wide spacing of childbirth (long birth interval), which enhances the nutritional status of women during pregnancy. Pregnant women who attended ≥4 ANC visits during pregnancy were more likely to take micronutrients compared with those who didn't attend ANC visits. This finding was in line with studies conducted in east African countries (25) and Bangladesh (53). This might be due to the fact that access to and utilization of ANC are essential for enhancing health and nutrition during pregnancy. As ANC provides a strategic platform for important healthcare functions, including health promotion and disease prevention, and 14 out of the 49 recommendations in the WHO ANC guideline are related to nutrition (54), adherence to ANC during pregnancy enhances the intake of micronutrients among pregnant women. During repeated ANC visits, pregnant women can get more information about the benefits of micronutrient intake through counseling. Furthermore, the odds of micronutrient intake were higher among pregnant women from urban areas. This might be due to the low uptake of micronutrients associated with the long distances to healthcare centers in rural areas. This might also be due to the lack of healthcare facilities in rural areas that contributed to lower rates of healthcare access among pregnant women, which has implications for accessing micronutrient supplementation. Pregnant women from a community with high media exposure were more likely to take micronutrients compared with their counterparts. Studies conducted in east African countries (25) and 26 sub-Saharan African countries (39) reported similar findings. Watching television, listening to radio, and reading newspapers could have a remarkable role in the dissemination of nutrition information and the improvement of knowledge for mothers, especially for less educated population groups and those who live in rural areas. Media-focused community-based health policies and practices, including mass media campaigns and continuing health education programs through the media, need to be considered to enhance micronutrient intake among pregnant women.

### Strengths and limitations of the study

This study's primary strength is its use of a large, nationally representative sample size from three SSA nations to identify determinants at the individual and community levels and assess pregnant women's micronutrient consumption status. Another strength of the current study is the application of sophisticated statistical models that take into account factors at the individual and community levels. The most recent DHS statistics from the three SSA nations were also used in the study. Policymakers may benefit from this research by increasing the consumption of micronutrients during pregnancy. The present investigation also has some limitations. Firstly, as we only selected three SSA nations in order to use the most recent data, the results might not apply to all of the region. Second, a causal relationship between the dependent variable and explanatory variables could not be established due to the cross-sectional nature of the study design. Thirdly, because the DHS dataset does not contain certain variables, such as medication supply and pregnant women's perception, these variables could not be addressed in this study. Lastly, because the DHS data relies on respondents’ self-reporting, recall bias may exist. Furthermore, the influence of regional income disparities on micronutrient intake is not analyzed.

## Conclusions

More than three-fourths of the study subjects were micronutrient-supplemented during their pregnancy. Women's age, educational status, marital status, working status, media exposure, birth interval, number of ANC visits, residence, and community media exposure were factors significantly associated with micronutrient intake. Therefore, improving women's education, disseminating nutrition information through media, providing more attention to young pregnant women who live in rural areas, increasing the number of ANC visits, and women's empowerment are recommended to increase micronutrient intake during pregnancy. Future researchers could better account for and address the impact of regional income disparities on micronutrient intake.

## Data Availability

Publicly available datasets were analyzed in this study. This data can be found here: https://dhsprogram.com/data/available-datasets.cfm.
